# Mitochondrial homeodynamics in ageing: mechanisms, resilience, and interventions

**DOI:** 10.1007/s10522-026-10506-0

**Published:** 2026-09-10

**Authors:** Piotr Paweł Chmielewski

**Affiliations:** Division of Anatomy, Department of Human Morphology and Embryology, Faculty of Medicine, Wroclaw Medical University, 6a Chałubińskiego Street, 50-368 Wrocław, Poland

**Keywords:** Ageing, Mitochondria, Mitophagy, Homeodynamics, Inflammageing, Exercise, Biomarkers

## Abstract

Mitochondria integrate bioenergetics, redox signalling, calcium handling, biosynthesis, apoptosis, and stress responses. Their contribution to ageing depends less on any single pathway than on the ability to sustain these functions through continuous maintenance, remodelling, and inter-organelle communication. This review proposes mitochondrial homeodynamics as a systems-level framework for that ability, which rests not on static preservation but on three linked capacities. Maintenance safeguards mitochondrial genome, proteome, and membrane integrity. Adaptation adjusts metabolism and remodels network and cristae architecture to match changing demand. Recovery restores function and reserve after challenge. These capacities emerge from mitochondrial quality control, network and cristae remodelling, biogenesis, mitophagy, retrograde stress signalling, and inter-organelle communication. So defined, mitochondrial dysfunction becomes a measurable loss of capacity rather than a descriptive category. Ageing erodes these capacities in tissue- and context-specific ways, which reduces physiological reserve, slows recovery after stress, and amplifies sterile inflammation. The mechanisms underlying these capacities, the biomarkers that report them, and the interventions proposed to preserve them are evaluated in turn. Exercise provides the strongest human evidence for coordinated mitochondrial and functional adaptation, whereas evidence for energy restriction, NAD+ precursors, mitophagy-supporting compounds, and mitochondria-targeted agents remains heterogeneous and endpoint-specific. No mitochondrial intervention has been shown to slow ageing or extend lifespan in healthy humans, and movement of a biomarker towards a younger reference value does not establish rejuvenation. Progress will require dynamic measures of maintenance, adaptation, and recovery, obtained in defined tissues and interpreted alongside clinically meaningful outcomes.

## Introduction

Age is the major risk factor for chronic disease and geriatric syndromes, but biological ageing varies across individuals, organs, cell types, and molecular systems (Partridge et al. 2018; López-Otín et al. 2023). Geroscience seeks modifiable processes that contribute to functional decline and multiple age-related diseases rather than treating each disorder as an isolated endpoint (Kennedy et al. 2014; Kritchevsky and Cummings 2025). A useful framework must therefore account for both progressive damage and differences in the capacity to maintain function during repeated metabolic, inflammatory, and environmental challenges.

Mitochondria integrate oxidative metabolism with redox signalling, calcium homeostasis, biosynthesis, apoptosis, innate immunity, and inter-organelle communication (Spinelli and Haigis 2018; Mottis et al. 2019). Their ageing phenotype is not a uniform decline in abundance or respiration, but a context-dependent combination of altered genome maintenance, proteostasis, network architecture, turnover, spatial distribution, metabolic flexibility, and stress responsiveness. Blood-cell respiration, for example, can increase with age and differs by sex (Phang et al. 2026). Mitochondrial homeodynamics is defined here as the systems property through which mitochondrial networks maintain integrity, adapt to demand, and recover after challenge. The term has been used previously for a nutrient-responsive switch between mitochondrial states during development (Zhang et al. 2025). It is used here in a related but distinct sense, for the capacity to sustain and restore such transitions across the lifespan. The concept emphasizes regulated flux, remodelling, and adaptive range rather than a fixed set point, and it follows the biogerontological view that ageing contracts the homeodynamic space despite persistent maintenance, repair, and defence (Rattan 2014). This formulation extends earlier accounts of mitochondrial resilience and stress adaptation (Picard and McEwen 2018; Sharma et al. 2019), and it organizes mitochondrial capacity into three interacting domains: maintenance, adaptation, and recovery. It does not designate a newly discovered pathway or a master mechanism of ageing.

This review examines the systems that support these domains, their alteration during ageing and inflammageing, and the evidence for candidate interventions. It proposes that precision geroscience should assess dynamic reserve and recovery alongside static biomarkers, without equating target engagement or partial normalization of mitochondrial function with slower ageing (Rattan 2020, 2026).

## Mechanisms underlying mitochondrial homeodynamic capacity

Mitochondrial homeodynamic capacity depends on coordinated surveillance, renewal, and signalling. Maintenance rests on quality-control systems that preserve genome and proteome integrity and sustain organelle distribution. Adaptation depends on the capacity of mitochondrial networks to remodel their structure and metabolism, activate transcriptional responses, and communicate with other cellular compartments during stress. Recovery requires restoration of adequate function without persistent inflammatory or cell-death signalling. Ageing can reduce this adaptive range despite continued maintenance and repair, even when basal measurements remain relatively preserved (Rattan 2014; Kauppila et al. 2017; Song et al. 2021).

### Mitochondrial genome maintenance and proteostasis

Mitochondrial genome integrity is sustained through replication, repair, nucleoid organization, selective turnover, and population-level buffering across many mtDNA copies. Heteroplasmic variants can remain functionally silent until their proportion exceeds mutation- and tissue-specific biochemical thresholds (Stewart and Chinnery 2015). Ageing is associated with the accumulation and clonal expansion of some mtDNA mutations, which arise predominantly from replication error rather than from oxidative damage (Kauppila et al. 2017), although mutation load alone does not determine mitochondrial failure. The phenotypic effect depends strongly on cell lineage, mutation type, energetic demand, and the efficiency with which dysfunctional genomes and organelles are constrained or removed. Recent work further links mitochondrial genome instability to inflammatory signalling, which extends the consequences of defective mtDNA maintenance beyond impaired oxidative phosphorylation (Bahat et al. 2025).

Proteostasis is equally important because most mitochondrial proteins are encoded in the nucleus, synthesized in the cytosol, imported into mitochondria, assembled into multiprotein complexes, and continuously surveyed. The human mitochondrial proteome exceeds one thousand proteins and is organized across subcompartments with distinct import, folding, and degradation requirements (Rath et al. 2021). Chaperones, ATP-dependent proteases, mitochondrial ribosome quality control, the mitochondrial unfolded protein response, and mitophagy form an integrated proteostasis network (Jensen and Jasper 2014; Suomalainen and Battersby 2018; Moehle et al. 2019; Song et al. 2021; Borlepawar et al. 2026). This network is particularly important in long-lived postmitotic cells, where cumulative proteotoxic stress cannot be diluted efficiently by cell division (Coleman et al. 2026). In primary human T cells, age-related loss of mitoribosome abundance and organization has recently been linked to impaired mitochondrial biogenesis and reduced cellular function, illustrating how organelle translation can become a cell-type-specific limiting node in human ageing (Chen et al. 2026).

### Network dynamics and spatial organization

Mitochondrial fusion and fission regulate content mixing, segregation of damaged regions, organelle distribution, and adaptation to energetic demand (Friedman and Nunnari 2014; Chan 2020). Neither fragmentation nor elongation is intrinsically beneficial. Functional relevance depends on the capacity to remodel appropriately. Experimental manipulation of either fission or fusion can extend lifespan under specific conditions in model organisms, which argues against treating one morphology as a universal marker of mitochondrial health (Sharma et al. 2019; Traa et al. 2024). The more informative property is network plasticity, which links morphology to turnover, bioenergetics, and stress recovery.

Studies of human skeletal muscle support the importance of structural organization. Mitochondrial fragmentation has been associated with lower physical capacity in older adults, and three-dimensional imaging has identified age-related changes in mitochondrial architecture that correlate with muscle characteristics (Goulding et al. 2025; Scudese et al. 2025). Changes in mitochondria-endoplasmic reticulum contacts can also occur early during age-related muscle dysfunction, with experimental evidence that regular exercise preserves aspects of contact-site organization (Allen et al. 2025). These observations do not establish that morphology is a causal biomarker of organismal ageing, but they show that mitochondrial structure and inter-organelle topology provide information that is not captured by abundance or resting respiration alone.

Spatial organization is especially critical in neurons and other highly polarized cells. Axonal and dendritic transport places mitochondria near sites of high ATP demand and calcium flux, while local quality-control pathways determine whether damaged organelles are repaired, returned to the soma, or removed (Misgeld and Schwarz 2017). With ageing, defects in transport or positioning can therefore produce local energetic failure before whole-cell mitochondrial measurements show major abnormalities. Similar principles apply to cardiomyocytes, skeletal muscle fibres, and stem-cell niches, where mitochondrial position is constrained by cellular architecture and functional demand (Ravindran and Gustafsson 2025). In the brain, mitochondrial dysfunction intersects with synaptic maintenance, proteostasis, inflammatory signalling, and neuronal vulnerability (Bartman et al. 2024), which makes regional and cell-type resolution particularly important when interpreting age-related mitochondrial phenotypes.

### Cristae organization and bioenergetic reserve

Mitochondrial architecture also extends to the inner membrane. Cristae provide a spatially organized environment for the respiratory-chain complexes and ATP synthase, and they support metabolite exchange and local electrochemical gradients (Spinelli and Haigis 2018; Chan 2020). Age-related changes in inner-membrane organization can therefore alter respiratory efficiency even when mitochondrial content is preserved. However, cristae density or shape should not be interpreted in isolation because structural remodelling can represent adaptation, compensation, or damage depending on tissue demand and the accompanying metabolic state. The relevant question is whether membrane organization supports sufficient reserve when ATP demand rises.

This distinction is important for human ageing studies. Bioenergetic reserve emerges from the interaction among membrane organization, calcium-sensitive metabolic activation, substrate availability, and network topology, as indicated by three-dimensional analyses of aged skeletal muscle and by evidence that impaired mitochondrial calcium uptake constrains muscle performance (Gherardi et al. 2025; Scudese et al. 2025). No single morphological feature can therefore serve as a universal readout of mitochondrial age. A stronger phenotype combines ultrastructure with respiratory reserve, metabolite handling, and tissue-level performance.

### Mitophagy, biogenesis, and mitochondrial turnover

Mitophagy removes damaged or superfluous mitochondria through PINK1-Parkin-dependent and receptor-mediated pathways, while basal mitophagy can proceed independently of PINK1 in several metabolically active tissues (McWilliams et al. 2018; Pickles et al. 2018). Mitophagy is not an isolated degradation process. It is coupled to mitochondrial fission, lysosomal competence, cellular energy status, and compensatory biogenesis. Experimental work in primary cells indicates that suppression of basal mitophagy can promote cellular ageing phenotypes, which supports a causal role for inadequate mitochondrial clearance in some contexts (Kelly et al. 2024).

Removal must be balanced by replacement. Mitochondrial biogenesis requires coordinated nuclear transcription, mtDNA replication, protein import, and organelle assembly, with PGC-1 coactivators and TFAM among its central regulators (Scarpulla 2008; Abu Shelbayeh et al. 2023). Work in Caenorhabditis elegans showed that coordination between mitophagy and biogenesis is necessary for organismal adaptation during ageing (Palikaras et al. 2015; Roussos et al. 2025). In mammalian tissues, the key translational issue is therefore turnover quality rather than maximal turnover rate. Excessive elimination without adequate replacement can reduce energetic capacity, whereas biogenesis without effective quality control can expand a dysfunctional pool.

The link between mitophagy and inflammatory control has become clearer. Impaired mitochondrial clearance can increase cytosolic mtDNA and activate cGAS-STING signalling, whereas enhanced mitophagy can restrain this inflammatory axis in ageing models (Jiménez-Loygorri et al. 2024). Skeletal muscle is a useful example because ageing, exercise, and disuse alter mitochondrial turnover in different directions and at different stages of the remodelling response (Kuznyetsova and Hood 2025). These findings support a dynamic interpretation in which mitophagy should be assessed together with lysosomal flux, biogenesis, and tissue function.

### Stress adaptation and inter-organelle communication

Mitochondria communicate continuously with the nucleus and with other organelles. Endoplasmic reticulum-mitochondria contact sites coordinate calcium transfer, lipid synthesis, organelle dynamics, and stress signalling. Lysosome-mitochondria contacts link nutrient sensing and organelle turnover, while contacts with peroxisomes and lipid droplets contribute to fatty-acid handling and redox balance (Murley and Nunnari 2016; Prinz et al. 2020; Wong et al. 2018). Metabolic kinases integrate these contact-site functions with cellular nutrient and stress signals, making inter-organelle communication an important component of ageing biology rather than a structural curiosity (Chowdhury et al. 2026).

Mitochondrial proteotoxic or bioenergetic stress can activate retrograde pathways, including the integrated stress response, which is relayed from the organelle by DELE1 (Fessler et al. 2020). At controlled levels, mitochondrial reactive oxygen species act as signals rather than solely as damaging by-products (Shadel and Horvath 2015; Sies and Jones 2020). This principle underlies mitohormesis, in which transient stress induces adaptation that increases subsequent resistance (Yun and Finkel 2014). The benefit of such signalling depends on stress intensity and duration, tissue, age, and baseline reserve.

Calcium handling is another homeodynamic node because mitochondrial calcium uptake links cellular work to oxidative metabolism. Recent mechanistic evidence indicates that mitochondrial calcium uptake can decline with ageing in skeletal muscle and contribute to reduced energetic performance (Gherardi et al. 2025). This observation fits the broader framework in which ageing can impair the ability to match mitochondrial output to fluctuating demand even when basal respiration is not uniformly depressed.

### Mitochondrial metabolites and nuclear signalling

Mitochondrial metabolism influences ageing biology not only via ATP production but also through metabolites that act as substrates, cofactors, or signals for nuclear and cytosolic enzymes. Changes in the NAD+/NADH ratio can alter redox reactions and NAD+-dependent signalling, while acetyl-CoA availability can influence protein acetylation and chromatin state. Tricarboxylic-acid-cycle intermediates such as alpha-ketoglutarate, succinate, and fumarate can affect dioxygenases and other regulatory enzymes. These relationships provide a mechanistic route by which mitochondrial state can reshape gene regulation, stress responses, and cell identity (Aon and Cortassa 2026; Covarrubias et al. 2021; Mottis et al. 2019).

The translational implication is more nuanced than the idea that one metabolite can reset biological age. Metabolite concentrations depend on compartment, flux, substrate use, oxygen availability, and cellular composition. The same metabolite can have different consequences across tissues or disease states. Thus, metabolomic and epigenomic changes can help define mitochondrial adaptation, but causal inference requires perturbation studies that show whether changing a specific mitochondrial process alters downstream gene regulation and ultimately preserves tissue function.

### Mitochondrial homeodynamics as an integrated systems property

**Fig. 1:**
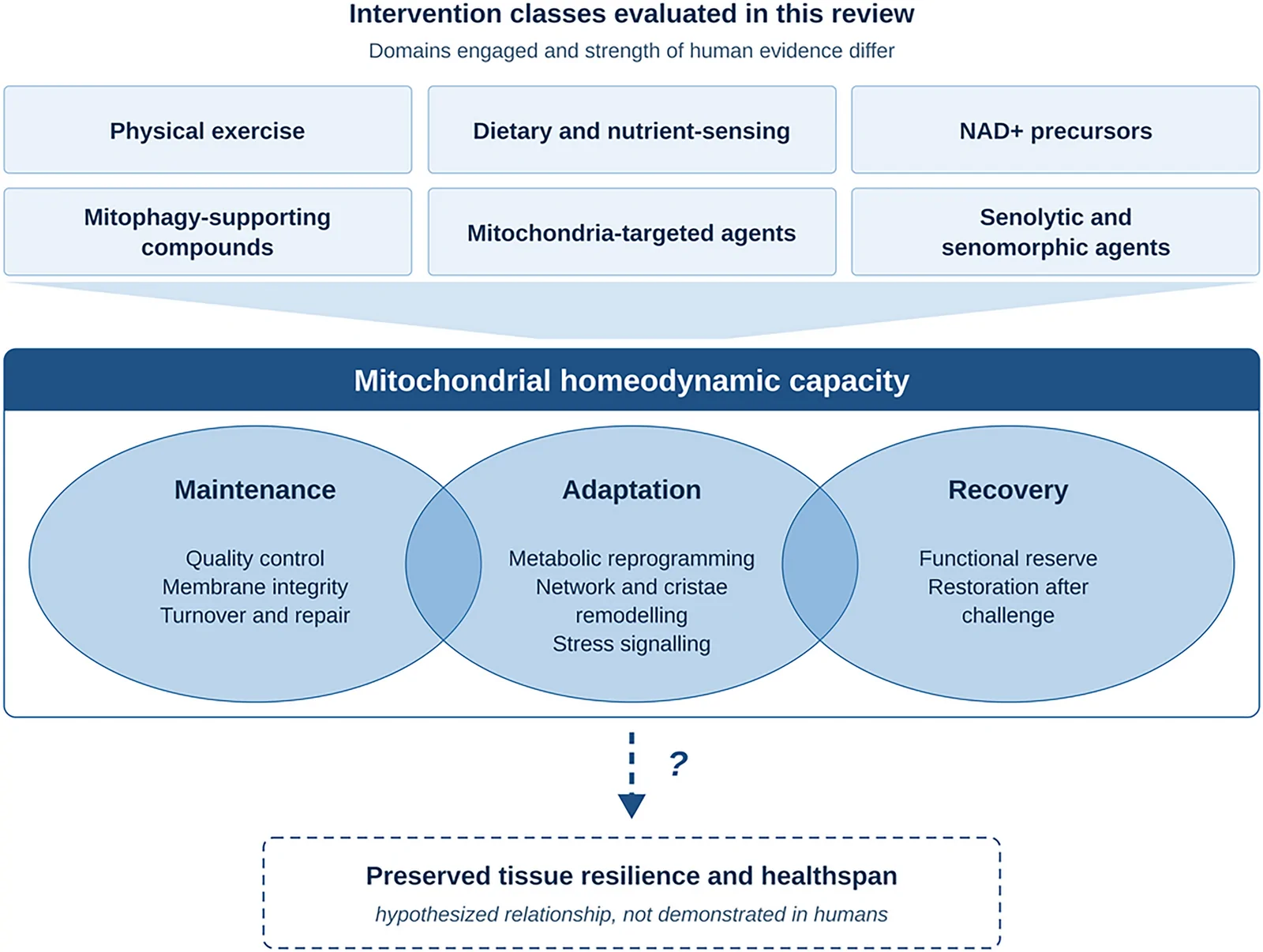
Mitochondrial homeodynamic capacity and its proposed relationship to tissue resilience. The central panel shows the three overlapping domains that together constitute mitochondrial homeodynamic capacity. Maintenance comprises genome and proteome quality control, membrane integrity, organelle turnover, and repair. Adaptation comprises metabolic reprogramming, network and cristae remodelling, and stress signalling. Recovery comprises the processes that restore function and reserve after challenge. The domains overlap because individual molecular processes contribute to more than one of them. The six boxes above list the intervention classes evaluated in this review, which differ in the domains they engage and in the strength of the supporting human evidence. The dashed arrow denotes a hypothesized relationship: whether preserving homeodynamic capacity slows functional decline or extends healthspan has not been tested in humans

The preceding mechanisms can be organized into three overlapping domains (Fig. 1). Maintenance comprises genome and proteome quality control, membrane integrity, turnover, and repair. Adaptation comprises metabolic reprogramming, network and cristae remodelling, and stress signalling. Recovery comprises the processes that restore function and reserve after challenge. Mitochondrial homeodynamic capacity is therefore a systems-level construct, not a single pathway, score, or youthful target state. Its value depends on reproducible measurement and association with functional outcomes. So defined, mitochondrial dysfunction is a measurable loss of capacity in one or more domains rather than a descriptive category.

The concept should not become a label for every favourable mitochondrial change. A valid homeodynamic interpretation must retain context, trade-offs, and the distinction between adaptive acute responses and harmful chronic activation (Rattan 2014). The safeguard against such circularity is to state the framework as predictions that can fail.

Three predictions follow. First, among individuals matched for age, sex, and resting mitochondrial capacity in a given tissue, the amplitude and rate of recovery after a standardized physiological challenge should predict subsequent functional decline, whereas resting measures alone should predict it less well or not at all. Second, an intervention that improves resting mitochondrial biomarkers without improving adaptation or recovery should not improve the functional outcomes attributed to its mitochondrial action, whereas an intervention that improves recovery kinetics should improve function even when resting measures are unchanged. Third, because the limiting domain differs across tissues and individuals, the response to a domain-specific intervention should be predictable from which domain is limiting at baseline, rather than idiosyncratic.

These predictions are testable with methods already applied in human skeletal muscle and blood, although standardized challenge protocols and validated recovery endpoints remain to be established (Ferrucci et al. 2020; Grevendonk et al. 2021). If measures of adaptation and recovery add no predictive information beyond resting mitochondrial phenotypes, and if intervention responses track biomarker change rather than restored adaptive range, then the homeodynamic framing carries no explanatory advantage over conventional accounts of mitochondrial decline and should be abandoned.

## Mitochondrial homeodynamics in ageing

Ageing does not produce a universal mitochondrial phenotype. It instead increases the probability that maintenance, adaptation, or recovery becomes limiting in a particular cell or tissue. Defects in mitophagy, proteostasis, biogenesis, network architecture, organelle communication, and metabolic flexibility vary in magnitude (Sun et al. 2016; Kauppila et al. 2017). Reduced reserve may therefore emerge only during exercise, fasting, infection, injury, or another challenge that requires rapid mitochondrial reconfiguration.

### Declining quality control and dynamic reserve

Long-lived cells are particularly sensitive to cumulative defects in quality control. In neurons, cardiomyocytes, and skeletal muscle, impaired proteostasis and organelle turnover can progressively increase the fraction of mitochondria that are structurally intact but functionally constrained (Affourtit and Carré 2024; Miwa et al. 2022; Moehle et al. 2019; Ravindran and Gustafsson 2025). Sarcopenia illustrates the interaction between mitochondrial decline, denervation, reduced physical activity, inflammation, impaired regeneration, and altered amino-acid metabolism (Zuo et al. 2025). At the same time, cross-sectional human studies show that mitochondrial phenotypes are not uniform across tissues, sexes, or age strata (Goulding et al. 2025; Phang et al. 2026). This variation should be treated as biological information rather than as measurement noise.

Stem cells provide a complementary example. Quiescent and activated stem cells occupy different metabolic states, and lineage commitment requires coordinated changes in mitochondrial dynamics, biogenesis, and oxidative metabolism (Khacho et al. 2016; Mohrin et al. 2015). Ageing-associated defects in these transitions can impair self-renewal and regenerative responses even when the baseline mitochondrial content of quiescent cells is low. Recent work in aged bone marrow further shows that mitochondrial abundance can identify haematopoietic stem-cell states with distinct self-renewal properties rather than functioning as a simple damage marker (Totani et al. 2025). The relevant phenotype is therefore the ability to transition between states rather than maximal mitochondrial output in one state.

### Tissue, sex, and life-course heterogeneity

A major limitation of broad claims about mitochondrial ageing is that different tissues face different constraints. Skeletal muscle must match large changes in energetic demand and preserve contractile function, the heart requires continuous oxidative output, neurons depend on long-distance organelle transport and local calcium buffering, immune cells undergo rapid metabolic reprogramming, and haematopoietic stem cells must balance quiescence with regenerative activation (Affourtit and Carré 2024; Chen et al. 2026; Coleman et al. 2026; Ravindran and Gustafsson 2025; Totani et al. 2025). A process that is limiting in one lineage may therefore be compensable in another. This heterogeneity argues against a single mitochondrial ageing score unless its components are explicitly tissue-aware.

Sex and life stage can also modify mitochondrial phenotypes and intervention responses. Human blood-cell respiration differs by sex across age, and exercise-associated adaptations depend on baseline activity, tissue, and physiological context (Bishop et al. 2025; Phang et al. 2026). Cross-sectional differences should be interpreted cautiously because they mix ageing effects with survivor selection, cohort effects, medication exposure, disease burden, and differences in cellular composition. Longitudinal designs and repeated challenge-response measurements are better suited to identifying trajectories of declining reserve. This point is especially relevant for precision geroscience, where biological heterogeneity is the object of study rather than a source of variance to be averaged away.

### The mitochondrial-inflammatory axis

Mitochondrial damage can amplify sterile inflammation when mitochondrial nucleic acids or other danger-associated signals enter inappropriate cellular compartments (Lin et al. 2022; Jindal et al. 2026). Cytosolic mtDNA can activate innate immune pathways, including cGAS-STING and the NLRP3 inflammasome (West et al. 2015; West and Shadel 2017; Zhong et al. 2018). In senescent cells, permeabilization of a minority of mitochondria can release mtDNA and reinforce the senescence-associated secretory phenotype (Victorelli et al. 2023). cGAS-STING signalling has also been implicated causally in age-related inflammation and neurodegeneration in experimental models (Gulen et al. 2023).

Recent evidence extends this axis beyond mtDNA. Mitochondrial genome instability caused by aberrant ribonucleotide incorporation can drive inflammatory signalling (Bahat et al. 2025), and mitochondrial RNA can accumulate in the cytosol of senescent cells and activate RNA-sensing pathways that sustain inflammatory secretion (Victorelli et al. 2025). Taken together, these observations support a broader concept in which defective mitochondrial compartmentalization and clearance can convert organelle stress into chronic innate immune activation. Mitophagy can interrupt this cycle by limiting the persistence and cytosolic exposure of damaged mitochondrial material (Jiménez-Loygorri et al. 2024).

Inflammation also acts in the opposite direction. Chronic cytokine exposure, altered nutrient signalling, and oxidative stress can impair mitochondrial biogenesis, dynamics, and quality-control pathways, which creates a self-reinforcing loop between mitochondrial dysfunction and inflammageing (Bektas et al. 2018; Franceschi et al. 2018; Miwa et al. 2022). This bidirectionality is important therapeutically because suppression of one inflammatory mediator does not necessarily restore the underlying mitochondrial maintenance defect, and correction of one mitochondrial parameter may not extinguish a sustained inflammatory programme.

### Intercellular and systemic mitochondrial signals

Mitochondrial quality control also extends beyond the boundaries of a single organelle. Mitochondria-derived vesicles can transport selected proteins, lipids, and other cargo to lysosomes, peroxisomes, or extracellular compartments, providing an additional route for organelle surveillance and intercellular communication (Ferrucci et al. 2024; König and McBride 2024). The significance of these vesicles in human ageing is still being defined, but they broaden the concept of mitochondrial homeodynamics from intracellular turnover to systemic signalling. Experimental approaches that transfer intact mitochondria or mitochondrial material are also being explored in regenerative medicine, although delivery, integration, immune compatibility, durability, and regulatory issues remain major barriers to clinical translation (Kubat et al. 2025).

Mitochondrial stress is also communicated systemically through secreted proteins. Activation of the integrated stress response (Fessler et al. 2020) induces GDF15 and FGF21, both of which circulate and are used as diagnostic markers of primary mitochondrial disease (Lehtonen et al. 2016; Yatsuga et al. 2015). Circulating GDF15 increases with age and has been associated with mortality, frailty, and functional decline in older cohorts (Conte et al. 2022; Wiklund et al. 2010). These proteins are currently the most accessible human readouts of mitochondrial stress signalling. They are not mitochondria-specific, however, because concentrations also respond to inflammation, renal function, malignancy, and common medications including metformin (Coll et al. 2020). They therefore report activation of a stress pathway rather than the capacity of the underlying domains, and they should be interpreted alongside tissue-level and challenge-based measures rather than as substitutes for them.

## Preserving and modulating mitochondrial homeodynamic capacity

Mitochondrial phenotypes are modifiable, but support for individual interventions is unequal. An intervention may change a molecular marker, improve an organ-specific function, or reduce a disease risk factor without slowing biological ageing. The sections below therefore distinguish mechanistic rationale, human functional evidence, and direct evidence for modification of ageing biology (Justice et al. 2016; Kritchevsky and Cummings 2025). They can also be read against the three domains. Mitophagy activators and mitochondria-targeted redox or membrane compounds act principally on maintenance, pharmacological nutrient-sensing modulators act principally on adaptation, and exercise and energy restriction, which are repeated physiological challenges, engage all three. Almost none of these interventions has been evaluated for effects on recovery after a standardized challenge, which is the gap the predictions above are intended to expose.

### Physical exercise

Exercise provides the strongest human evidence for coordinated improvement in mitochondrial function and physical capacity. In skeletal muscle, training activates AMPK- and calcium-sensitive signalling, PGC-1-coordinated transcription, mitochondrial protein synthesis, antioxidant defences, and turnover (Bishop et al. 2025; Grevendonk et al. 2021). Exercise is a repeated multidimensional challenge followed by recovery, not a single-target therapy. Its benefits reflect integrated adaptation across tissues and cannot be inferred from one downstream pathway.

Responses vary by modality, intensity, baseline health, age, and tissue. Endurance training generally increases oxidative capacity, while resistance training supports muscle mass and strength. Human studies indicate that exercise can preserve mitochondrial network architecture and organelle contact sites (Allen et al. 2025; Scudese et al. 2025), and physical activity can modify immune-cell metabolism in older adults (Hazeldine et al. 2025). These effects support functional adaptation but do not establish reversal of mitochondrial ageing.

Frailty, pain, disability, and multimorbidity can limit structured exercise in older adults, supporting the development of adjunctive strategies. However, reproducing one exercise-responsive pathway cannot substitute for the integrated mechanical, metabolic, vascular, immune, neural, and behavioural stimulus. Candidate interventions should therefore be judged by safe functional benefit rather than resemblance to selected molecular effects of exercise (Rattan 2020).

### Dietary and nutrient-sensing interventions

Energy and nutrient restriction engage AMPK, mTOR, insulin-IGF signalling, autophagy, and stress responses (de Cabo and Mattson 2019; Longo and Anderson 2022). Caloric restriction extends lifespan in several model organisms. In the CALERIE trial, sustained caloric restriction improved cardiometabolic risk factors and induced metabolic adaptation, but the study did not test human lifespan (Kraus et al. 2019; Redman et al. 2018). Fasting-mimicking and time-restricted approaches can modify metabolic markers, although evidence for durable age-related clinical outcomes remains incomplete (Wei et al. 2017).

Nutrient composition can influence ageing pathways independently of energy intake. Restriction of specific amino acids or protein alters metabolism, frailty, and lifespan in rodents, with effects modified by sex and life stage (Green et al. 2023; Richardson et al. 2021; Yeh et al. 2024). These findings should not be extrapolated directly to older adults because frailty, sarcopenia, and malnutrition can make sustained restriction harmful. Nutritional strategies must preserve lean tissue, bone health, and adequacy.

### NAD+ metabolism and mitochondrial signalling

NAD+ links redox metabolism with sirtuin activity, DNA repair, and stress signalling, and its metabolism changes with age (Covarrubias et al. 2021). Nicotinamide riboside and nicotinamide mononucleotide increase NAD+-related metabolites in humans, but effects on metabolic, vascular, inflammatory, and functional outcomes are inconsistent (Elhassan et al. 2019; Yoshino et al. 2021). Biochemical target engagement establishes biological activity rather than geroprotection, and NAD+ precursors remain unproven as interventions that slow human ageing.

### Mitophagy-supporting and autophagy-related strategies

Urolithin A induces mitophagy in experimental systems and produces molecular evidence of target engagement in humans (Andreux et al. 2019; Ryu et al. 2016). A randomized trial in older adults reported improvements in selected muscle-endurance outcomes with mitochondrial biomarker changes, but the magnitude and durability of clinical benefit remain uncertain (Liu et al. 2022). These findings support further study, not claims of organismal rejuvenation.

Spermidine promotes autophagy and longevity in model organisms, and higher dietary intake is associated with lower mortality (Eisenberg et al. 2009; Kiechl et al. 2018). A randomized trial of spermidine supplementation in older adults with subjective cognitive decline did not improve its primary cognitive endpoint, and no reproducible geroprotective effect has been established in humans (Schwarz et al. 2022). Translation of mitophagy modulation requires evidence of tissue benefit without maladaptive mitochondrial depletion or other long-term effects.

### Mitochondria-targeted redox, membrane, and metabolic approaches

Mitochondria-targeted compounds test whether selected redox or bioenergetic defects are modifiable in humans. MitoQ improved endothelial function in a small study of healthy older adults (Rossman et al. 2018). Elamipretide acutely increased skeletal-muscle ATP production, but a longer heart-failure trial did not improve its primary endpoint (Butler et al. 2020; Roshanravan et al. 2021). These results show why short-term target engagement cannot be generalized to organismal ageing, particularly when reactive species also mediate adaptive signalling.

Metformin and mTOR inhibition affect nutrient sensing rather than mitochondria alone. Both have extensive mechanistic support, but neither is a validated anti-ageing therapy for healthy humans. Metformin attenuated some exercise-induced mitochondrial adaptations in older adults (Konopka et al. 2019). mTOR inhibition has improved selected immune outcomes, although results vary across endpoints (Kell et al. 2026; Mannick et al. 2021). The metformin finding illustrates a problem that a homeodynamic reading makes explicit. An agent can engage a plausible target while reducing the adaptive response to a stimulus of established benefit, so combinations of interventions require testing for interference rather than assumed additivity.

### Senescence, inflammageing, and indirect mitochondrial rescue

Senescent cells can alter neighbouring tissues through the senescence-associated secretory phenotype and mitochondrial nucleic-acid signalling (Gorgoulis et al. 2019; Victorelli et al. 2023). Senolytic and senomorphic strategies may therefore reduce inflammatory pressure on mitochondrial maintenance (Kirkland and Tchkonia 2020). Early human studies show feasibility in selected diseases but not anti-ageing efficacy (Justice et al. 2019). Effects will probably be tissue specific because senescent cells also contribute to wound repair and tumour suppression in some contexts.

### Translation, safety, and intervention windows

Interventions must be evaluated in the physiological context of older adults. Frailty, multimorbidity, polypharmacy, altered pharmacokinetics, reduced renal or hepatic reserve, and nutritional vulnerability can alter both benefit and risk. Suppression of growth signalling, changes in substrate availability, or increased organelle turnover may have different consequences in middle age than in late life with sarcopenia or acute illness. Preclinical lifespan extension therefore cannot substitute for human safety and functional evidence (Justice et al. 2016; Rattan 2020).

Intervention timing also matters. Some mechanisms may be most modifiable before extensive tissue damage, whereas pathways governing inflammation, immunity, or recovery may remain actionable later. Human trials show that pathways can be engaged in older adults, but organ-specific effects cannot be generalized to whole-body ageing (Farr et al. 2024; Mannick et al. 2021). Development should proceed from target engagement to organ function and validated healthspan outcomes, with safety assessed at each stage.

## Mitochondrial homeodynamics in precision geroscience

Precision geroscience requires measures that separate chronological age from variation in reserve, vulnerability, and recovery (Ahadi et al. 2020; Ferrucci et al. 2020). Multi-omic and plasma-proteomic studies show that ageing trajectories differ among individuals and organs (Oh et al. 2023). Cross-species analyses can help determine whether mitochondrial phenotypes are upstream, downstream, or parallel to broader ageing programmes (Tyshkovskiy et al. 2026). This heterogeneity argues against assuming a universal mitochondrial age or intervention response.

Static measures such as resting respiration, mitochondrial copy number, or a circulating marker are informative but cannot fully capture a response property. Standardized challenge-based phenotyping could quantify the amplitude and kinetics of adaptation and recovery, using measures such as respiratory reserve, substrate switching, mitophagic flux, redox recovery, and functional performance. Such measures require validation for reproducibility, tissue specificity, sex effects, and prediction of clinical outcomes (Grevendonk et al. 2021; Phang et al. 2026).

Tissue access remains a major limitation. Muscle biopsy permits detailed phenotyping but is invasive, while blood-cell assays may not represent brain, heart, or muscle. Circulating mtDNA, extracellular vesicles, proteomic signatures, metabolites, and stress-induced mitokines such as GDF15 are influenced by inflammation, exercise, infection, injury, and pre-analytical handling. Multimodal frameworks should therefore integrate molecular signals with organ function and longitudinal trajectories rather than rely on one surrogate (Ferrucci et al. 2020; Oh et al. 2023).

Operationally, maintenance can be assessed through genome and proteome integrity, turnover, and organelle quality. Adaptation can be assessed through metabolic reprogramming, stress signalling, and network remodelling, and recovery through the time required to restore function after challenge. This is a hypothesis-generating framework, not a validated composite biomarker. Its value should be tested by whether it improves prediction of resilience, frailty, mobility loss, or multimorbidity beyond established measures (Ferrucci et al. 2020; Kritchevsky and Cummings 2025). Testing the predictions set out above depends on precisely these measurements, which is at present the main practical obstacle to evaluating the framework.

### Causal attribution in geroscience interventions

The central challenge in geroscience is causal attribution. If an intervention improves walking speed, glucose control, respiration, and inflammatory markers, the changes may arise from a shared upstream mechanism, parallel mechanisms, or secondary effects of increased activity (Kennedy et al. 2014; Kritchevsky and Cummings 2025).

Mediation studies are therefore needed to determine whether mitochondrial changes contribute to clinical benefit or merely accompany it, especially for pleiotropic interventions such as exercise, energy restriction, and nutrient-sensing modulation. The term geroscience intervention should describe a hypothesis, not confer therapeutic status in advance (Rattan 2020).

Exceptional longevity is consistent with preserved resilience but does not establish mitochondrial homeodynamics as its cause (Andersen et al. 2012). Likewise, mitochondrial biomarker improvement is not rejuvenation without durable evidence of better function or reduced vulnerability. Even healthspan requires an explicit operational definition (Rattan 2026). Conservative terminology improves testability and credibility.

Cell-state manipulation, organelle transfer, and mitochondrial engineering can test constraints inaccessible to lifestyle interventions, but remain predominantly preclinical or early translational (Kubat et al. 2025). Development requires tissue targeting, durable integration, immune compatibility, genomic and epigenomic stability, assessment of tumour risk, and benefit beyond short-term molecular endpoints. These requirements determine whether an experimental manipulation can credibly preserve human function.

### Trial design: from target engagement to healthspan

Clinical trials should state the evidentiary step being tested. Early studies can establish feasibility, safety, target engagement, and short-term molecular or physiological effects, but not modification of ageing. Subsequent trials should determine whether target engagement produces durable changes in organ function, resilience, or validated outcomes across more than one age-related domain (Justice et al. 2016; Kritchevsky and Cummings 2025). The interpretation should not exceed the endpoint measured.

Dynamic outcomes may be especially informative because mitochondrial reserve often becomes apparent only under challenge. Recovery after exercise, metabolic stress, vaccination, or another standardized challenge could complement resting biomarkers. These outcomes should be paired with established measures of mobility, cognition, immune function, cardiometabolic health, frailty, and adverse events and analysed within a prespecified causal framework (Ferrucci et al. 2020).

Because lifespan trials are rarely feasible initially, longer studies may use incident multimorbidity, disability-free survival, major age-related disease events, or validated measures of intrinsic capacity and frailty. Molecular biomarkers can add mechanistic information, but should serve as primary or co-primary outcomes only after analytical validity, biological interpretation, and responsiveness to intervention have been established (Kritchevsky and Cummings 2025).

More mitochondrial activity is not invariably beneficial. Excessive substrate oxidation, persistent stress signalling, uncontrolled biogenesis, or defective removal can be maladaptive. The objective is regulated flexibility rather than maximization. Mitochondrial homeodynamics therefore describes the capacity to change appropriately and recover sufficiently, not a mandate to maximize respiration, NAD+, mitophagy, biogenesis, or any other single parameter.

## Research priorities and unresolved questions

Five questions should define the next phase of mitochondrial geroscience:

- (1) Which measurements reproducibly capture mitochondrial maintenance, adaptation, and recovery in humans?
- (2) Do these measurements predict functional decline independently of chronological age and established risk factors?
- (3) Which evidentiary claim is a given trial designed to support: target engagement, organ-specific functional improvement, modification of an ageing-related risk process, or reduction in clinical events? These outcomes are related but not equivalent.
- (4) How do sex, ancestry, tissue, baseline fitness, nutritional status, and comorbidity modify mitochondrial phenotypes and intervention responses (Ahadi et al. 2020; Phang et al. 2026)
- (5) What do negative results and failed extrapolations reveal about the validity and limits of the proposed model (Rattan 2024)

Experimental design must also distinguish restoration from compensation. Function may recover through alternative substrates, increased mitochondrial mass, altered calcium handling, or changes in cell composition without correction of the initiating lesion. Conversely, a molecular defect may improve while another component continues to limit organ function. Studies should therefore combine molecular assays with physiology and clinically meaningful outcomes (Ferrucci et al. 2020; Justice et al. 2016).

## Conclusions

Mitochondrial ageing is better described as erosion of coordinated maintenance, adaptation, and recovery than as uniform loss of ATP production or a simple increase in oxidative stress. Genome maintenance, proteostasis, network dynamics, turnover, calcium handling, stress signalling, and inter-organelle communication jointly determine mitochondrial responses to demand. Their failure can reduce tissue reserve and amplify inflammageing, but mitochondrial changes may be initiating, amplifying, compensatory, or consequential depending on tissue and context. Exercise provides the strongest human evidence that mitochondrial and physiological reserve remain modifiable in later life. Nutritional, NAD+-directed, mitophagy-supporting, mitochondria-targeted, and broader geroscience interventions have plausible targets but less consistent evidence for durable clinical benefit. None has established extended human lifespan or generalized slowing of ageing. Mitochondrial homeodynamics will be useful only if operationalized through dynamic, tissue-aware measures that improve prediction of resilience, frailty, and multimorbidity. The translational aim is to preserve adaptive capacity and function without disrupting compensatory biology, not to restore every mitochondrial feature to a youthful value.

## Data Availability

No datasets were generated or analysed during the current study.

## References

[CR1] Abu Shelbayeh O, Arroum T, Morris S, Busch KB (2023) PGC-1α is a master regulator of mitochondrial lifecycle and ROS stress response. Antioxidants (Basel) 12:1075 10.3390/antiox12051075 PMC10215733 37237941

[CR2] Affourtit C, Carré JE (2024) Mitochondrial involvement in sarcopenia. Acta Physiol 240:e14107 10.1111/apha.14107 38304924

[CR3] Ahadi S, Zhou W, Schüssler-Fiorenza Rose SM, Sailani MR, Contrepois K, Avina M et al (2020) Personal aging markers and ageotypes revealed by deep longitudinal profiling. Nat Med 26:83–90 10.1038/s41591-019-0719-5 PMC7301912 31932806

[CR4] Allen RJ, Kronemberger A, Shi Q, Pope RM, Cuadra-Muñoz E, Son W et al (2025) Altered relaxation and mitochondria-endoplasmic reticulum contacts precede major (mal)adaptations in aging skeletal muscle and are prevented by exercise. Aging Cell 24:e70137 10.1111/acel.70137 PMC12419855 40586781

[CR5] Andersen SL, Sebastiani P, Dworkis DA, Feldman L, Perls TT (2012) Health span approximates life span among many supercentenarians: compression of morbidity at the approximate limit of life span. J Gerontol A Biol Sci Med Sci 67:395–405 10.1093/gerona/glr223 PMC3309876 22219514

[CR6] Andreux PA, Blanco-Bose W, Ryu D, Burdet F, Ibberson M, Aebischer P et al (2019) The mitophagy activator urolithin A is safe and induces a molecular signature of improved mitochondrial and cellular health in humans. Nat Metab 1:595–603 10.1038/s42255-019-0073-4 32694802

[CR7] Aon MA, Cortassa S (2026) Systems biology of aging, metabolism, and mitochondria. Annu Rev Biophys. 10.1146/annurev-biophys-021424-011852 41666034

[CR8] Bahat A, Milenkovic D, Cors E, Barnett M, Niftullayev S, Katsalifis A et al (2025) Ribonucleotide incorporation into mitochondrial DNA drives inflammation. Nature 647:726–734 10.1038/s41586-025-09541-7 PMC12629987 40993386

[CR9] Bartman S, Coppotelli G, Ross JM (2024) Mitochondrial dysfunction: a key player in brain aging and diseases. Curr Issues Mol Biol 46:1987–2026 10.3390/cimb46030130 PMC10969191 38534746

[CR10] Bektas A, Schurman SH, Sen R, Ferrucci L (2018) Aging, inflammation and the environment. Exp Gerontol 105:10–18 10.1016/j.exger.2017.12.015 PMC5909704 29275161

[CR11] Bishop DJ, Lee MJC, Picard M (2025) Exercise as mitochondrial medicine: how does the exercise prescription affect mitochondrial adaptations to training? Annu Rev Physiol 87:107–129 10.1146/annurev-physiol-022724-104836 39656953

[CR12] Borlepawar A, Neu M, Ma Z, Deshpande A, Bühringer H, Hajieva P, Frey N, Rangrez AY (2026) Emerging perspectives in proteostasis: bridging mechanisms and therapeutics for human diseases. Signal Transduct Target Ther 11:224 10.1038/s41392-026-02714-4 PMC13246805 42259777

[CR13] Butler J, Khan MS, Anker SD, Fonarow GC, Kim RJ, Nodari S et al (2020) Effects of elamipretide on left ventricular function in patients with heart failure with reduced ejection fraction: the PROGRESS-HF phase 2 trial. J Card Fail 26:429–437 10.1016/j.cardfail.2020.02.001 32068002

[CR14] Chan DC (2020) Mitochondrial dynamics and its involvement in disease. Annu Rev Pathol 15:235–259 10.1146/annurev-pathmechdis-012419-032711 31585519

[CR15] Chen J, Su L, Pan B, Sang J, Li L, Huang J et al (2026) Cryoelectron tomography reveals an age-related decline in mitoribosomes that contributes to T cell dysfunction in older individuals. Proc Natl Acad Sci U S A 123:e2608102123 10.1073/pnas.2608102123 PMC13438555 42525521

[CR16] Chowdhury MR, Jeon JH, Chanda D (2026) Metabolic kinases as regulators of inter-organelle communication in aging and age-related diseases. Aging Cell 25:e70624 10.1111/acel.70624 PMC13333541 42402668

[CR17] Coleman RA, Johnson ME, Konopka A, Chew YL (2026) Proteostasis, disease and the ageing neuron: compartmental complexity in non-renewing cells. Ageing Res Rev 118:103073 10.1016/j.arr.2026.103073 41765108

[CR18] Coll AP, Chen M, Taskar P, Rimmington D, Patel S, Tadross JA et al (2020) GDF15 mediates the effects of metformin on body weight and energy balance. Nature 578:444–448 10.1038/s41586-019-1911-y PMC7234839 31875646

[CR19] Conte M, Giuliani C, Chiariello A, Iannuzzi V, Franceschi C, Salvioli S (2022) GDF15, an emerging key player in human aging. Ageing Res Rev 75:101569 10.1016/j.arr.2022.101569 35051643

[CR20] Covarrubias AJ, Perrone R, Grozio A, Verdin E (2021) NAD+ metabolism and its roles in cellular processes during ageing. Nat Rev Mol Cell Biol 22:119–141 10.1038/s41580-020-00313-x PMC7963035 33353981

[CR21] de Cabo R, Mattson MP (2019) Effects of intermittent fasting on health, aging, and disease. N Engl J Med 381:2541–2551 10.1056/NEJMra1905136 31881139

[CR22] Eisenberg T, Knauer H, Schauer A, Büttner S, Ruckenstuhl C, Carmona-Gutierrez D et al (2009) Induction of autophagy by spermidine promotes longevity. Nat Cell Biol 11:1305–1314 10.1038/ncb1975 19801973

[CR23] Elhassan YS, Kluckova K, Fletcher RS, Schmidt MS, Garten A, Doig CL et al (2019) Nicotinamide riboside augments the aged human skeletal muscle NAD+ metabolome and induces transcriptomic and anti-inflammatory signatures. Cell Rep 28:1717-1728.e6 10.1016/j.celrep.2019.07.043 PMC6702140 31412242

[CR24] Farr JN, Atkinson EJ, Achenbach SJ, Volkman TL, Tweed AJ, Vos SJ et al (2024) Effects of intermittent senolytic therapy on bone metabolism in postmenopausal women: a phase 2 randomized controlled trial. Nat Med 30:2605–2612 10.1038/s41591-024-03096-2 PMC11705617 38956196

[CR25] Ferrucci L, Gonzalez-Freire M, Fabbri E, Simonsick E, Tanaka T, Moore Z et al (2020) Measuring biological aging in humans: a quest. Aging Cell 19:e13080 10.1111/acel.13080 PMC6996955 31833194

[CR26] Ferrucci L, Guerra F, Bucci C, Marzetti E, Picca A (2024) Mitochondria break free: mitochondria-derived vesicles in aging and associated conditions. Ageing Res Rev 102:102549 10.1016/j.arr.2024.102549 PMC12155116 39427885

[CR27] Fessler E, Eckl EM, Schmitt S, Mancilla IA, Meyer-Bender MF, Hanf M et al (2020) A pathway coordinated by DELE1 relays mitochondrial stress to the cytosol. Nature 579:433–437 10.1038/s41586-020-2076-4 PMC7116715 32132706

[CR28] Franceschi C, Garagnani P, Morsiani C, Conte M, Santoro A, Grignolio A, Monti D, Capri M, Salvioli S (2018) The continuum of aging and age-related diseases: common mechanisms but different rates. Front Med 5:61 10.3389/fmed.2018.00061 PMC5890129 29662881

[CR29] Friedman JR, Nunnari J (2014) Mitochondrial form and function. Nature 505:335–343 10.1038/nature12985 PMC4075653 24429632

[CR30] Gherardi G, Weiser A, Bermont F, Migliavacca E, Brinon B, Jacot GE et al (2025) Mitochondrial calcium uptake declines during aging and is directly activated by oleuropein to boost energy metabolism and skeletal muscle performance. Cell Metab 37:477-495.e11 10.1016/j.cmet.2024.10.021 39603237

[CR31] Gorgoulis V, Adams PD, Alimonti A, Bennett DC, Bischof O, Bishop C et al (2019) Cellular senescence: defining a path forward. Cell 179:813–827 10.1016/j.cell.2019.10.005 31675495

[CR32] Goulding RP, Charlton B, Breedveld E, van der Laan M, Strating A, Noort W et al (2025) Skeletal muscle mitochondrial fragmentation predicts age-associated decline in physical capacity. Aging Cell 24:e14386 10.1111/acel.14386 PMC11822651 39630001

[CR33] Green CL, Trautman ME, Chaiyakul K, Jain R, Alam YH, Babygirija R et al (2023) Dietary restriction of isoleucine increases healthspan and lifespan of genetically heterogeneous mice. Cell Metab 35:1976-1995.e6 10.1016/j.cmet.2023.10.005 PMC10655617 37939658

[CR34] Grevendonk L, Connell NJ, McCrum C, Fealy CE, Bilet L, Bruls YMH et al (2021) Impact of aging and exercise on skeletal muscle mitochondrial capacity, energy metabolism, and physical function. Nat Commun 12:4773 10.1038/s41467-021-24956-2 PMC8346468 34362885

[CR35] Gulen MF, Samson N, Keller A, Schwabenland M, Liu C, Glück S et al (2023) cGAS-STING drives ageing-related inflammation and neurodegeneration. Nature 620:374–380 10.1038/s41586-023-06373-1 PMC10412454 37532932

[CR36] Hazeldine J, Withnall E, Llibre A, Duggal NA, Lord JM, Sardeli AV (2025) Physical activity modifies the metabolic profile of CD4+ and CD8+ T-cell subtypes at rest and upon activation in older adults. Aging Cell 24:e70104 10.1111/acel.70104 PMC12266771 40400170

[CR37] Jensen MB, Jasper H (2014) Mitochondrial proteostasis in the control of aging and longevity. Cell Metab 20:214–225 10.1016/j.cmet.2014.05.006 PMC4274350 24930971

[CR38] Jiménez-Loygorri JI, Villarejo-Zori B, Viedma-Poyatos Á, Zapata-Muñoz J, Benítez-Fernández R, Frutos-Lisón MD et al (2024) Mitophagy curtails cytosolic mtDNA-dependent activation of cGAS/STING inflammation during aging. Nat Commun 15:830 10.1038/s41467-024-45044-1 PMC10821893 38280852

[CR39] Jindal D, Chopra V, Garg K, Sharma S (2026) Mitochondrial DNA-mediated cGAS-STING activation and its crosstalk with NLRP3 inflammasome in chronic obstructive pulmonary disease. Cytokine Growth Factor Rev 90:12–25 10.1016/j.cytogfr.2026.05.001 42143499

[CR40] Justice J, Miller JD, Newman JC, Hashmi SK, Halter J, Austad SN et al (2016) Frameworks for proof-of-concept clinical trials of interventions that target fundamental aging processes. J Gerontol A Biol Sci Med Sci 71:1415–1423 10.1093/gerona/glw126 PMC5055651 27535966

[CR41] Justice JN, Nambiar AM, Tchkonia T, LeBrasseur NK, Pascual R, Hashmi SK et al (2019) Senolytics in idiopathic pulmonary fibrosis: results from a first-in-human, open-label, pilot study. EBioMedicine 40:554–563 10.1016/j.ebiom.2018.12.052 PMC6412088 30616998

[CR42] Kauppila TES, Kauppila JHK, Larsson NG (2017) Mammalian mitochondria and aging: an update. Cell Metab 25:57–71 10.1016/j.cmet.2016.09.017 28094012

[CR43] Kell L, Jones EJ, Gharahdaghi N, Wilkinson DJ, Smith K, Atherton PJ et al (2026) Rapamycin exerts its geroprotective effects in the ageing human immune system by enhancing resilience against DNA damage. Aging Cell 25:e70364 10.1111/acel.70364 PMC12794675 41524558

[CR44] Kelly G, Kataura T, Panek J, Ma G, Salmonowicz H, Davis A et al (2024) Suppressed basal mitophagy drives cellular aging phenotypes that can be reversed by a p62-targeting small molecule. Dev Cell 59:1924-1939.e7 10.1016/j.devcel.2024.04.020 38897197

[CR45] Kennedy BK, Berger SL, Brunet A, Campisi J, Cuervo AM, Epel ES et al (2014) Geroscience: linking aging to chronic disease. Cell 159:709–713 10.1016/j.cell.2014.10.039 PMC4852871 25417146

[CR46] Khacho M, Clark A, Svoboda DS, Azzi J, MacLaurin JG, Meghaizel C et al (2016) Mitochondrial dynamics impacts stem cell identity and fate decisions by regulating a nuclear transcriptional program. Cell Stem Cell 19:232–247 10.1016/j.stem.2016.04.015 27237737

[CR47] Kiechl S, Pechlaner R, Willeit P, Notdurfter M, Paulweber B, Willeit K et al (2018) Higher spermidine intake is linked to lower mortality: a prospective population-based study. Am J Clin Nutr 108:371–380 10.1093/ajcn/nqy102 29955838

[CR48] Kirkland JL, Tchkonia T (2020) Senolytic drugs: from discovery to translation. J Intern Med 288:518–536 10.1111/joim.13141 PMC7405395 32686219

[CR49] König T, McBride HM (2024) Mitochondrial-derived vesicles in metabolism, disease, and aging. Cell Metab 36:21–35 10.1016/j.cmet.2023.11.014 38171335

[CR50] Konopka AR, Laurin JL, Schoenberg HM, Reid JJ, Castor WM, Wolff CA et al (2019) Metformin inhibits mitochondrial adaptations to aerobic exercise training in older adults. Aging Cell 18:e12880 10.1111/acel.12880 PMC6351883 30548390

[CR51] Kraus WE, Bhapkar M, Huffman KM, Pieper CF, Krupa Das S, Redman LM, Villareal DT, Rochon J, Roberts SB, Ravussin E, Holloszy JO, Fontana L, CALERIE Investigators (2019) 2 years of calorie restriction and cardiometabolic risk (CALERIE): exploratory outcomes of a multicentre, phase 2, randomised controlled trial. Lancet Diabetes Endocrinol 7:673–683 10.1016/S2213-8587(19)30151-2 PMC6707879 31303390

[CR52] Kritchevsky SB, Cummings SR (2025) Geroscience: a translational review. JAMA 334:1094–1102 10.1001/jama.2025.11289 40773213

[CR53] Kubat GB, Picone P, Tuncay E, Aryan L, Girgenti A, Palumbo L et al (2025) Biotechnological approaches and therapeutic potential of mitochondria transfer and transplantation. Nat Commun 16:5709 10.1038/s41467-025-61239-6 PMC12217326 40593725

[CR54] Kuznyetsova A, Hood DA (2025) Mitophagy in skeletal muscle: impact of ageing, exercise and disuse. Exp Physiol. 10.1113/EP093041 PMC13395031 41259646

[CR55] Lehtonen JM, Forsström S, Bottani E, Viscomi C, Baris OR, Isoniemi H et al (2016) FGF21 is a biomarker for mitochondrial translation and mtDNA maintenance disorders. Neurology 87:2290–2299 10.1212/WNL.0000000000003374 PMC5270510 27794108

[CR56] Lin MM, Liu N, Qin ZH, Wang Y (2022) Mitochondrial-derived damage-associated molecular patterns amplify neuroinflammation in neurodegenerative diseases. Acta Pharmacol Sin 43:2439–2447 10.1038/s41401-022-00879-6 PMC9525705 35233090

[CR57] Liu S, D’Amico D, Shankland E, Bhayana S, Garcia JM, Aebischer P, Rinsch C, Singh A, Marcinek DJ (2022) Effect of urolithin A supplementation on muscle endurance and mitochondrial health in older adults: a randomized clinical trial. JAMA Netw Open 5:e2144279 10.1001/jamanetworkopen.2021.44279 PMC8777576 35050355

[CR58] Longo VD, Anderson RM (2022) Nutrition, longevity and disease: from molecular mechanisms to interventions. Cell 185:1455–1470 10.1016/j.cell.2022.04.002 PMC9089818 35487190

[CR59] López-Otín C, Blasco MA, Partridge L, Serrano M, Kroemer G (2023) Hallmarks of aging: an expanding universe. Cell 186:243–278 10.1016/j.cell.2022.11.001 36599349

[CR60] Mannick JB, Teo G, Bernardo P, Quinn D, Russell K, Klickstein L et al (2021) Targeting the biology of ageing with mTOR inhibitors to improve immune function in older adults: phase 2b and phase 3 randomised trials. Lancet Healthy Longev 2:e250–e262 10.1016/S2666-7568(21)00062-3 PMC8102040 33977284

[CR61] McWilliams TG, Prescott AR, Montava-Garriga L, Ball G, Singh F, Barini E et al (2018) Basal mitophagy occurs independently of PINK1 in mouse tissues of high metabolic demand. Cell Metab 27:439-449.e5 10.1016/j.cmet.2017.12.008 PMC5807059 29337137

[CR62] Misgeld T, Schwarz TL (2017) Mitostasis in neurons: maintaining mitochondria in an extended cellular architecture. Neuron 96:651–666 10.1016/j.neuron.2017.09.055 PMC5687842 29096078

[CR63] Miwa S, Kashyap S, Chini E, von Zglinicki T (2022) Mitochondrial dysfunction in cell senescence and aging. J Clin Invest 132:e158447 10.1172/JCI158447 PMC9246372 35775483

[CR64] Moehle EA, Shen K, Dillin A (2019) Mitochondrial proteostasis in the context of cellular and organismal health and aging. J Biol Chem 294:5396–5407 10.1074/jbc.TM117.000893 PMC6462515 29622680

[CR65] Mohrin M, Shin J, Liu Y, Brown K, Luo H, Xi Y, Haynes CM, Chen D (2015) Stem cell aging. A mitochondrial UPR-mediated metabolic checkpoint regulates hematopoietic stem cell aging. Science 347:1374–1377 10.1126/science.aaa2361 PMC4447312 25792330

[CR66] Mottis A, Herzig S, Auwerx J (2019) Mitocellular communication: shaping health and disease. Science 366:827–832 10.1126/science.aax3768 31727828

[CR67] Murley A, Nunnari J (2016) The emerging network of mitochondria-organelle contacts. Mol Cell 61:648–653 10.1016/j.molcel.2016.01.031 PMC5554544 26942669

[CR68] Oh HS, Rutledge J, Nachun D, Pálovics R, Abiose O, Moran-Losada P, Channappa D, Urey DY, Kim K, Sung YJ et al (2023) Organ aging signatures in the plasma proteome track health and disease. Nature 624:164–172 10.1038/s41586-023-06802-1 PMC10700136 38057571

[CR69] Palikaras K, Lionaki E, Tavernarakis N (2015) Coordination of mitophagy and mitochondrial biogenesis during ageing in *C. elegans*. Nature 521:525–528 10.1038/nature14300 25896323

[CR70] Partridge L, Deelen J, Slagboom PE (2018) Facing up to the global challenges of ageing. Nature 561:45–56 10.1038/s41586-018-0457-8 30185958

[CR71] Phang HJ, Bergstrom J, Keri B, Heimler SR, Dozier S, Scandalis LM et al (2026) Blood cell mitochondrial respiration increases with age and varies by sex in healthy adults. Aging Cell 25:e70387 10.1111/acel.70387 PMC12823460 41566162

[CR72] Picard M, McEwen BS (2018) Psychological stress and mitochondria: a conceptual framework. Psychosom Med 80:126–140 10.1097/PSY.0000000000000544 PMC5901651 29389735

[CR73] Pickles S, Vigié P, Youle RJ (2018) Mitophagy and quality control mechanisms in mitochondrial maintenance. Curr Biol 28:R170–R185 10.1016/j.cub.2018.01.004 PMC7255410 29462587

[CR74] Prinz WA, Toulmay A, Balla T (2020) The functional universe of membrane contact sites. Nat Rev Mol Cell Biol 21:7–24 10.1038/s41580-019-0180-9 PMC10619483 31732717

[CR75] Rath S, Sharma R, Gupta R, Ast T, Chan C, Durham TJ et al (2021) MitoCarta3.0: an updated mitochondrial proteome now with sub-organelle localization and pathway annotations. Nucleic Acids Res 49:D1541–D1547 10.1093/nar/gkaa1011 PMC7778944 33174596

[CR76] Rattan SIS (2014) Molecular gerontology: from homeodynamics to hormesis. Curr Pharm des 20:3036–3039 10.2174/13816128113196660708 24079765

[CR77] Rattan SIS (2020) Naive extrapolations, overhyped claims and empty promises in ageing research and interventions need avoidance. Biogerontology 21:415–421 10.1007/s10522-019-09851-0 31773357

[CR78] Rattan SIS (2024) Seven knowledge gaps in modern biogerontology. Biogerontology 25:1–8 10.1007/s10522-023-10089-0 38206540

[CR79] Rattan SIS (2026) Lifespan stops at death, but when does healthspan stop? Biogerontology 27:78 10.1007/s10522-026-10424-1 41903027

[CR80] Ravindran R, Gustafsson ÅB (2025) Mitochondrial quality control in cardiomyocytes: safeguarding the heart against disease and ageing. Nat Rev Cardiol 22:798–813 10.1038/s41569-025-01142-1 40113864

[CR81] Redman LM, Smith SR, Burton JH, Martin CK, Il’yasova D, Ravussin E (2018) Metabolic slowing and reduced oxidative damage with sustained caloric restriction support the rate of living and oxidative damage theories of aging. Cell Metab 27:805–815 10.1016/j.cmet.2018.02.019 PMC5886711 29576535

[CR82] Richardson NE, Konon EN, Schuster HS, Mitchell AT, Boyle C, Rodgers AC et al (2021) Lifelong restriction of dietary branched-chain amino acids has sex-specific benefits for frailty and lifespan in mice. Nat Aging 1:73–86 10.1038/s43587-020-00006-2 PMC8009080 33796866

[CR83] Roshanravan B, Liu SZ, Ali AS, Shankland EG, Goss C, Amory JK et al (2021) In vivo mitochondrial ATP production is improved in older adult skeletal muscle after a single dose of elamipretide in a randomized trial. PLoS ONE 16:e0253849 10.1371/journal.pone.0253849 PMC8282018 34264994

[CR84] Rossman MJ, Santos-Parker JR, Steward CAC, Bispham NZ, Cuevas LM, Rosenberg HL et al (2018) Chronic supplementation with a mitochondrial antioxidant (MitoQ) improves vascular function in healthy older adults. Hypertension 71:1056–1063 10.1161/HYPERTENSIONAHA.117.10787 PMC5945293 29661838

[CR85] Roussos A, Kitopoulou K, Borbolis F, Ploumi C, Gianniou DD, Li Z, He H, Tsakiri E, Borland H, Kostakis IK, Samiotaki M, Trougakos IP, Bohr VA, Palikaras K (2025) Urolithin Α modulates inter-organellar communication via calcium-dependent mitophagy to promote healthy ageing. Autophagy 21:3097–3122 10.1080/15548627.2025.2561073 PMC12758194 40944367

[CR86] Ryu D, Mouchiroud L, Andreux PA, Katsyuba E, Moullan N, Nicolet-dit-Félix AA et al (2016) Urolithin A induces mitophagy and prolongs lifespan in *C. elegans* and increases muscle function in rodents. Nat Med 22:879–888 10.1038/nm.4132 27400265

[CR87] Scarpulla RC (2008) Transcriptional paradigms in mammalian mitochondrial biogenesis and function. Physiol Rev 88:611–638 10.1152/physrev.00025.2007 18391175

[CR88] Schwarz C, Benson GS, Horn N, Wurdack K, Grittner U, Schilling R, Märschenz S, Köbe T, Hofer SJ, Magnes C et al (2022) Effects of spermidine supplementation on cognition and biomarkers in older adults with subjective cognitive decline: a randomized clinical trial. JAMA Netw Open 5:e2213875 10.1001/jamanetworkopen.2022.13875 PMC9136623 35616942

[CR89] Scudese E, Marshall AG, Vue Z, Exil V, Rodriguez BI, Demirci M et al (2025) 3D mitochondrial structure in aging human skeletal muscle: insights into MFN-2-mediated changes. Aging Cell 24:e70054 10.1111/acel.70054 PMC12266761 40285369

[CR90] Shadel GS, Horvath TL (2015) Mitochondrial ROS signaling in organismal homeostasis. Cell 163:560–569 10.1016/j.cell.2015.10.001 PMC4634671 26496603

[CR91] Sharma A, Smith HJ, Yao P, Mair WB (2019) Causal roles of mitochondrial dynamics in longevity and healthy aging. EMBO Rep 20:e48395 10.15252/embr.201948395 PMC6893295 31667999

[CR92] Sies H, Jones DP (2020) Reactive oxygen species (ROS) as pleiotropic physiological signalling agents. Nat Rev Mol Cell Biol 21:363–383 10.1038/s41580-020-0230-3 32231263

[CR93] Song J, Herrmann JM, Becker T (2021) Quality control of the mitochondrial proteome. Nat Rev Mol Cell Biol 22:54–70 10.1038/s41580-020-00300-2 33093673

[CR94] Spinelli JB, Haigis MC (2018) The multifaceted contributions of mitochondria to cellular metabolism. Nat Cell Biol 20:745–754 10.1038/s41556-018-0124-1 PMC6541229 29950572

[CR95] Stewart JB, Chinnery PF (2015) The dynamics of mitochondrial DNA heteroplasmy: implications for human health and disease. Nat Rev Genet 16:530–542 10.1038/nrg3966 26281784

[CR96] Sun N, Youle RJ, Finkel T (2016) The mitochondrial basis of aging. Mol Cell 61:654–666 10.1016/j.molcel.2016.01.028 PMC4779179 26942670

[CR97] Suomalainen A, Battersby BJ (2018) Mitochondrial diseases: the contribution of organelle stress responses to pathology. Nat Rev Mol Cell Biol 19:77–92 10.1038/nrm.2017.66 28792006

[CR98] Totani H, Matsumura T, Yokomori R, Umemoto T, Takihara Y, Yang C et al (2025) Mitochondria-enriched hematopoietic stem cells exhibit elevated self-renewal capabilities, thriving within the context of aged bone marrow. Nat Aging 5:831–847 10.1038/s43587-025-00828-y 40050412

[CR99] Traa A, Keil A, AlOkda A, Jacob-Tomas S, Tamez González AA, Zhu S et al (2024) Overexpression of mitochondrial fission or mitochondrial fusion genes enhances resilience and extends longevity. Aging Cell 23:e14262 10.1111/acel.14262 PMC11464124 38953684

[CR100] Tyshkovskiy A, Kholdina D, Davitadze M, Molière A, Moldakozhayev A, Tongu Y et al (2026) Universal transcriptomic hallmarks of mammalian ageing and mortality. Nature 654:173–188 10.1038/s41586-026-10542-3 PMC13233323 42203874

[CR101] Victorelli S, Salmonowicz H, Chapman J, Martini H, Vizioli MG, Riley JS et al (2023) Apoptotic stress causes mtDNA release during senescence and drives the SASP. Nature 622:627–636 10.1038/s41586-023-06621-4 PMC10584674 37821702

[CR102] Victorelli S, Eppard M, Woo SH, Everts SPA, Martini H, Pirius N et al (2025) Mitochondrial RNA cytosolic leakage drives the SASP. Nat Commun 16:10992 10.1038/s41467-025-66159-z PMC12705736 41398033

[CR103] Wei M, Brandhorst S, Shelehchi M, Mirzaei H, Cheng CW, Budniak J et al (2017) Fasting-mimicking diet and markers/risk factors for aging, diabetes, cancer, and cardiovascular disease. Sci Transl Med 9:eaai8700 10.1126/scitranslmed.aai8700 PMC6816332 28202779

[CR104] West AP, Shadel GS (2017) Mitochondrial DNA in innate immune responses and inflammatory pathology. Nat Rev Immunol 17:363–375 10.1038/nri.2017.21 PMC7289178 28393922

[CR105] West AP, Khoury-Hanold W, Staron M, Tal MC, Pineda CM, Lang SM et al (2015) Mitochondrial DNA stress primes the antiviral innate immune response. Nature 520:553–557 10.1038/nature14156 PMC4409480 25642965

[CR106] Wiklund FE, Bennet AM, Magnusson PKE, Eriksson UK, Lindmark F, Wu L et al (2010) Macrophage inhibitory cytokine-1 (MIC-1/GDF15): a new marker of all-cause mortality. Aging Cell 9:1057–1064 10.1111/j.1474-9726.2010.00629.x PMC4139960 20854422

[CR107] Wong YC, Ysselstein D, Krainc D (2018) Mitochondria-lysosome contacts regulate mitochondrial fission via RAB7 GTP hydrolysis. Nature 554:382–386 10.1038/nature25486 PMC6209448 29364868

[CR108] Yatsuga S, Fujita Y, Ishii A, Fukumoto Y, Arahata H, Kakuma T et al (2015) Growth differentiation factor 15 as a useful biomarker for mitochondrial disorders. Ann Neurol 78:814–823 10.1002/ana.24506 PMC5057301 26463265

[CR109] Yeh CY, Chini LCS, Davidson JW, Garcia GG, Gallagher MS, Freichels IT et al (2024) Late-life protein or isoleucine restriction impacts physiological and molecular signatures of aging. Nat Aging 4:1760–1771 10.1038/s43587-024-00744-7 PMC11672203 39604703

[CR110] Yoshino M, Yoshino J, Kayser BD, Patti GJ, Franczyk MP, Mills KF et al (2021) Nicotinamide mononucleotide increases muscle insulin sensitivity in prediabetic women. Science 372:1224–1229 10.1126/science.abe9985 PMC8550608 33888596

[CR111] Yun J, Finkel T (2014) Mitohormesis. Cell Metab 19:757–766 10.1016/j.cmet.2014.01.011 PMC4016106 24561260

[CR112] Zhang J, Liu S, Li Y, Xu G, Deng H, King-Jones K et al (2025) Nutrient status alters developmental fates via a switch in mitochondrial homeodynamics. Nat Commun 16:1258 10.1038/s41467-025-56528-z PMC11787341 39893174

[CR113] Zhong Z, Liang S, Sanchez-Lopez E, He F, Shalapour S, Lin XJ et al (2018) New mitochondrial DNA synthesis enables NLRP3 inflammasome activation. Nature 560:198–203 10.1038/s41586-018-0372-z PMC6329306 30046112

[CR114] Zuo X, Zhao R, Wu M, Wang Y, Wang S, Tang K et al (2025) Multi-omic profiling of sarcopenia identifies disrupted branched-chain amino acid catabolism as a causal mechanism and therapeutic target. Nat Aging 5:419–436 10.1038/s43587-024-00797-8 39910243

